# Big Data and Pediatric Acute Kidney Injury: The Promise of Electronic Health Record Systems

**DOI:** 10.3389/fped.2019.00536

**Published:** 2020-01-14

**Authors:** Scott M. Sutherland

**Affiliations:** Division of Nephrology, Department of Pediatrics, Stanford University, Stanford, CA, United States

**Keywords:** acute kidney injury (AKI), pediatrics, big data and analytics, electronic health record (EHR), outcomes

## Abstract

Over the last decade, our understanding of acute kidney injury (AKI) has evolved considerably. The development of a consensus definition standardized the approach to identifying and investigating AKI in children. As a result, pediatric AKI epidemiology has been refined and the consequences of renal injury are better established. Similarly, “big data” methodologies experienced a dramatic evolution and maturation, leading the critical care community to explore potential AKI/big data synergies. One such concept with tremendous potential is electronic health record (EHR) enabled informatics. Much of the promise surrounding these approaches is due to the unique position of the EHR which sits at the intersection of data accumulation and care delivery. EHR data is generated simply via the provision of routine clinical care and should be considered “big” from the standpoint of volume, variety, and velocity as a myriad of diverse elements accumulate rapidly in real time, spontaneously generating an immense dataset. This massive dataset interfaces directly with providers which creates tremendous opportunity. AKI can be diagnosed more accurately, AKI-related care can be optimized, and subsequent outcomes can be improved. Although applying big data concepts to the EHR has proven more challenging than originally thought, we have seen much success and continue to explore its potential. In this review article, we will discuss the EHR in the context of big data concepts, describe approaches applied to date, examine the challenges surrounding optimal application, and explore future directions.

## Introduction

Acute kidney injury (AKI) has become a common complication amongst hospitalized children (1–3). Studies utilizing modern, consensus definitions report a prevalence of ~5 and 25% in children receiving acute and critical care, respectively (3, 4). The frequency with which AKI occurs is of particular concern given its outcome implications. AKI has been associated with greater mortality, longer lengths of hospital and intensive care unit (ICU) stay, and the subsequent development of chronic kidney disease (CKD) (3, 5, 6). Recently, the critical care and nephrology communities have standardized the definition of AKI, culminating in the Kidney Disease: Improving Global Outcomes (KDIGO) guidelines which identify AKI events based on increasing serum creatinine and/or decreasing urine output (UOP) (7). With this development, AKI can be identified consistently across practice environments, data sets, and health care platforms. In parallel, we have seen substantial growth in the adoption of electronic health records (EHRs) as well as the development of innovative clinical informatics methods (8–10). While the establishment of a uniform approach to AKI identification and the evolution of healthcare informatics are not causally related, the temporal relationship has created unique opportunities for AKI research and care improvement.

Many of the aforementioned informatics techniques and methodologies have been categorized as “big data,” a relatively novel concept to healthcare practitioners. Big data (or Big Data) is defined by the Oxford English Dictionary as, “data of a very large size, typically to the extent that its manipulation and management present significant logistical challenges; (also) the branch of computing involving such data (11).” Based on this definition, it is relatively easy to see the connection between big data and the EHR. The data contained within the EHR is “big” from the standpoint of volume (amount of data present), velocity (speed at which new data is generated), and variety (number of different types of data) (12–14). With regard to AKI, this means that the EHR contains all creatinine and UOP data for all patients affiliated with a particular organization, accrues new creatinine and UOP data in real time, and possesses a near-infinite number of AKI related data elements which are created and stored through the provision of routine patient care.

Thus, the EHR and its data create a unique opportunity (14, 15). The ability to accurately identify AKI events within a clinical platform allows AKI to be explored retrospectively, investigated prospectively, and studied for quality improvement or benchmarking purposes. Although the application of big data approaches to AKI research and care has proven more challenging than originally thought, we continue to explore refined, clinically applicable synergies. The goal of this manuscript is to consider the EHR in the context of big data concepts, appraise the approaches applied to date, examine the challenges surrounding optimal application, and explore future directions.

## AKI Identification and Diagnosis

**Figure 1:**
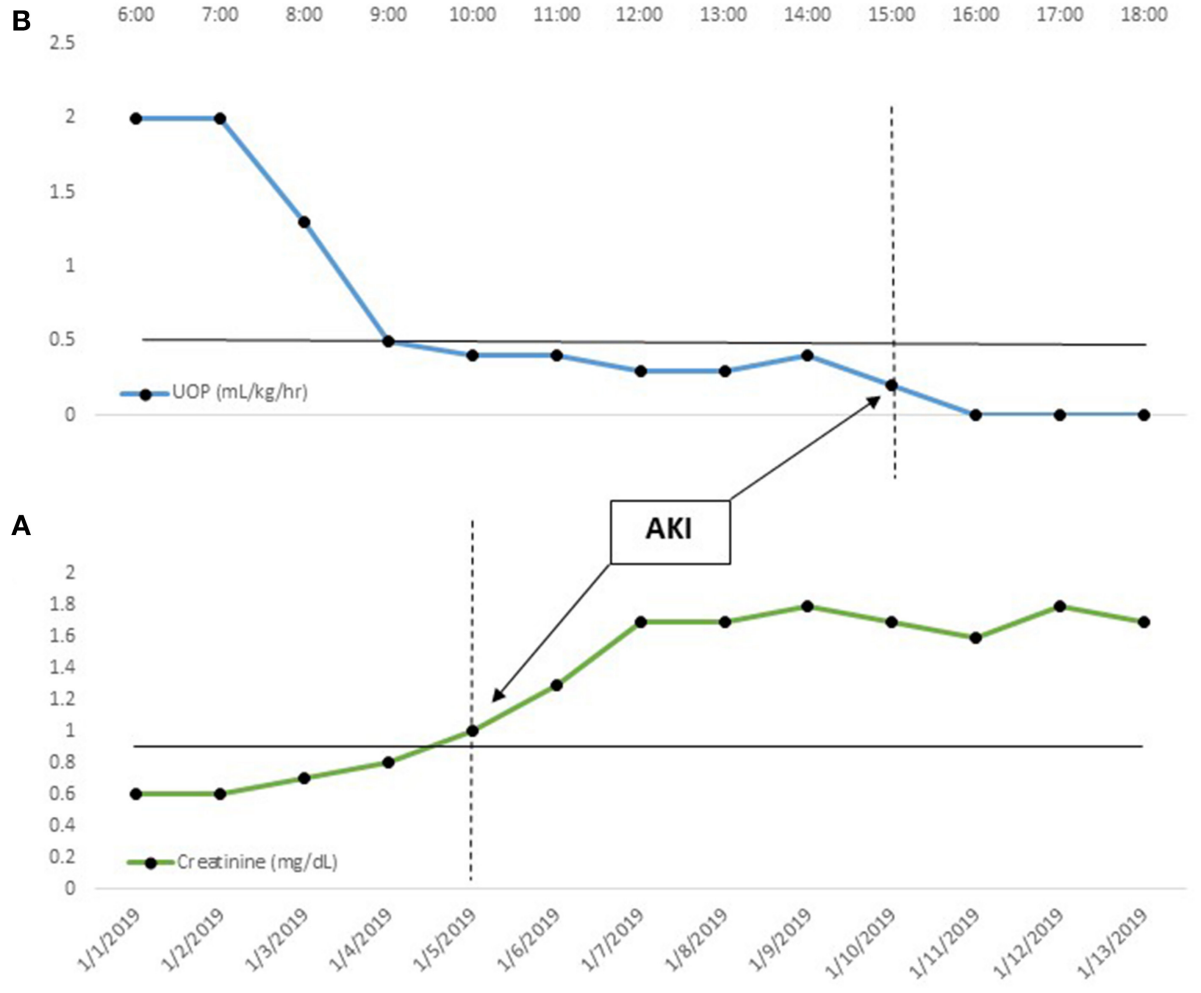
Automated, Real-time AKI Identification. In this figure, temporal creatinine (daily) and urine output (hourly) trends are displayed. In **(A)**, the creatinine gradually increases from a baseline of 0.6 mg/dL, meeting AKI criteria (serum creatinine > 1.5× baseline) on 1/5/19. Likewise, in **(B)**, the patient develops progressive oliguria, meeting AKI criteria (UOP < 0.5 mL/kg/h for 6 h) at 15:00. In both cases, the ability to detect the threshold value upon documentation allows real-time diagnosis. This, in turn, opens up a myriad of big data AKI solutions.

The cornerstone of EHR-enabled, big data AKI research and quality improvement is the ability to precisely diagnosis AKI events (14, 15). EHR data allows us, in a relatively straightforward manner, to identify AKI in real time by applying the KDIGO serum creatinine and/or UOP criteria (Figure 1) (7, 16, 17). For example, as creatinines become available, they may be compared to all prior creatinine values for that patient, and AKI may be diagnosed when the relative change threshold is met (Figure 1A). Serum creatinine results are discrete data which accumulate with an associated date and time; this, in turn, allows application of the full temporal components of the KDIGO definition. The same principles can be applied to the UOP criteria (Figure 1B). UOP is recorded hourly in milliliters (mL) and dividing this value by the patient's weight in kilograms (kg) generates a per-kg-per-unit-time rate (mL/kg/h).

Although automated, real-time AKI identification is technically feasible, aspects of the definition itself can be challenging to operationalize. One example is baseline creatinine determination. Setting each patient's baseline is important as it forms the basis for relative change determinations. Several approaches exist, each of which poses a different big data concern (16, 18–22). If available, a pre-admission creatinine may be used for the baseline. Many studies employing this approach have selected the lowest creatinine value available from the preceding 3–6 months. Outside of neonates and children under 3–6 months of age, creatinine is not likely to undergo a physiologic change in that timeframe. Unfortunately, prior creatinines are often unavailable within the EHR; this may be due to patients receiving ambulatory care in other health systems or it is possible, especially in pediatrics, that no prior creatinine has ever been obtained. When an actual value is not available, many have recommended using the admission creatinine as the baseline. This approach, while simple and effective from an informatics standpoint, will miss community acquired AKI which is manifest on admission; previously published studies suggest that this may underestimate the AKI burden by a third (20). Alternatively, a baseline creatinine can be estimated by back-calculating using a presumed creatinine clearance (CrCl). Studies in adults and children have tended to assume the CrCl to be 75 and 100–120 mL/min/1.73 m^2^, respectively (18, 20, 21). This will capture community acquired AKI but misclassifies patients with chronic kidney disease (CKD) as having AKI. In adults, where CKD is highly prevalent, this approach can overestimate AKI incidence by AKI 50% (20). Technically, this method does requires a computation, adding complexity any automated AKI identification diagnostic tool. Furthermore, nearly all estimating equations require data which are unreliably available within the EHR (i.e., height and ethnicity). A final option for patients without a known baseline, is to apply an age-based normative creatinine value. In this scenario, a population-based serum creatinine is assigned to each patient based on their demographic characteristics (23, 24). Each of these potential solutions have been validated and, ultimately, the approach applied should reflect the goals of the diagnostic tool.

The UOP criteria also pose challenges, however, in this case the issues tend to be related to EHR limitations rather than definitional shortcomings. The most substantial issue is urine volumes are not obtained with the rigor or regularity of creatinine. Outside of the ICUs, very few children have indwelling urinary catheters capable of providing hourly data. As a result, these patients may not have urine data recorded for hours. In children, urine may be documented only as a void count, without giving a specific volume. Given the short temporal interval set by KDIGO, this could result in patients with normal renal function being inaccurately diagnosed with AKI. Secondly, EHRs tend to aggregate intake and output data at static 8–12 h intervals which coincide with nursing shifts. The UOP criteria, however, necessitate a dynamic approach which utilizes a rolling 6–24 h window. Processing a rolling calculation for each patient across an institution may pose resource, computational, and logistic challenges. Despite these potential issues, the ability to accurately diagnose AKI in real-time is technically feasible and this capacity unlocks numerous big-data approaches to AKI care. To fully realize its potential, however, a standardized solution and approach to the aforementioned problems must be adopted by the critical care nephrology community.

While no established approach yet exists, information is available to inform our approach. With regard to the creatinine criteria, most agree that a previously obtained creatinine should be used as the baseline value if it is available (3, 14, 15, 25). If computational resources are unlimited, one could determine and use the mean serum creatinine from the prior 12 months (25, 26). However, using the creatinine most proximal to the admission is a simpler solution which has demonstrated similar efficacy (26). If a creatinine is not available, given the relatively low incidence of CKD in children, using an imputed value as the baseline is a reasonable approach. Most studies to date have back calculated the imputed serum creatinine using an estimated creatinine clearance of 120 mL/min/1.73 m^2^. However, using an age based normative value may be equally effective and will reduce computational requirements (3, 24); either approach should be considered valid. With regard to UOP, it is important to note that studies in adults and children demonstrate that some children meet only the UOP criteria for AKI; non-application of these KDIGO thresholds may underestimate AKI incidence (3, 27). Thus, if possible, the UOP criteria should be integrated into any diagnostic tool if possible. One reasonable compromise is to utilize the 12 h summative information in its static form to generate a volume-per-kg-per-hour rate. Although this is not as accurate as a dynamic window and will miss oliguria of <12 h duration, it is a simple way to implement the UOP criteria that will capture a larger portion of the true AKI population.

## Predicting Acute Kidney Injury Events

**Figure 2:**
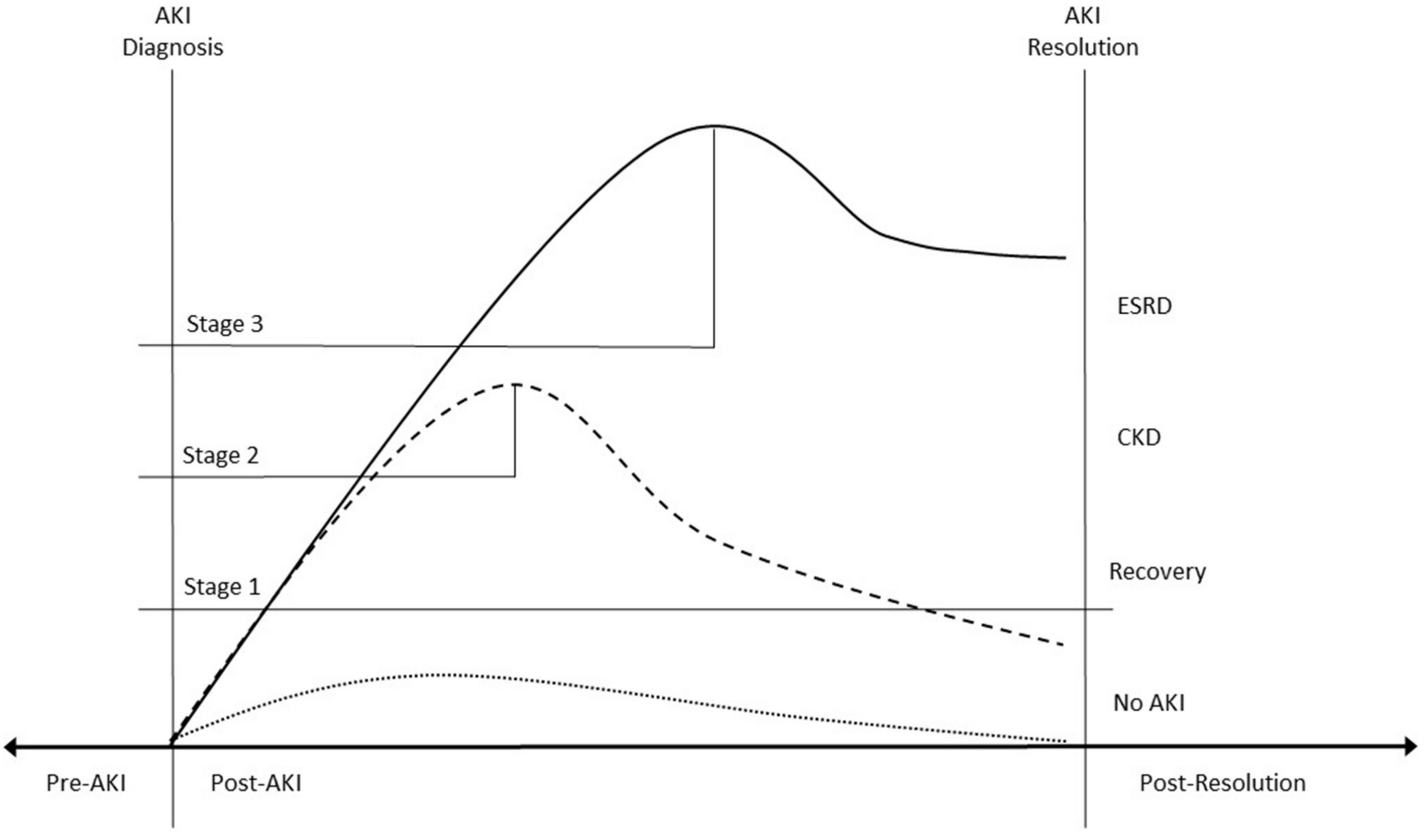
Impact of Big-Data AKI Interventions. Real-time AKI diagnosis temporally separates EHR data into pre-illness and post-illness categories. The pre-AKI data can be used for predictive analytics. The ability to accurately identify patients at high AKI risk allows preventative strategies to be employed. An episode of Stage 2 AKI (dashed line) could be completely prevented, flattening the creatinine trajectory (dotted line). After AKI is diagnosed, real time notification allows providers to modify care with the goal of mitigating disease severity. Appropriate interventions might result in a patient developing Stage 2 (dashed line) rather than Stage 3 AKI (unbroken line). This, in turn, might improve long term (AKI resolution) outcomes. Regardless, once the AKI has resolved, the ability to identify these patients accurately, allows them to be “tagged” and followed whether they developed ESRD, CKD, or experienced full recovery.

Once AKI is accurately diagnosed in real-time, a number of EHR-enabled interventions become viable. One of the most exciting prospects is AKI prediction—detecting events before they occur. AKI events can be temporally anchored within the EHR which creates a pre-disease phase of care containing the data which accumulated prior to AKI. High-content, high-throughput techniques can be applied to this data to identify a pre-AKI signal which, in turn, can help discriminate between patients at low and high risk for AKI. The ability to predict AKI risk in this way may have dramatic impact as there are not currently treatments for AKI once it has developed (28–30). As patients at high risk are identified, care can be modified and preventative and harm avoidance strategies can be implemented (Figure 2) (31–36).

AKI prediction was the subject of the 15th Acute Dialysis Quality Initiative (ADQI) conference (13, 37–39). This conference highlighted several aspects related to AKI prediction and risk stratification which impact our ability to fully realize the potential of this big data approach. This consensus statement noted that at the time of publication, almost all AKI prediction models had employed a “supervised” approach, meaning that potential predictors were chosen *a priori* based upon their association with AKI in prior studies (40–45). While certainly statistically sound, these approaches do not take full advantage of big data informatics methods. “Unsupervised” techniques identify predictors without oversight or prior prejudice. Although they represent a departure from more traditional model building approaches, the use of these innovative, dynamic techniques are necessary to completely optimize the use of EHR data (13).

Since the 15th ADQI conference, a number of studies examining AKI prediction models have been performed. An excellent systematic review of prognostic models was published in 2017 (46). Hodgson et al. identified 53 models designed to predict hospital acquired AKI, 11 of which met their inclusion criteria. Although the area under the receiver operative curve (AUROC) ranged from 0.71 to 0.8 in the model derivation populations, AUROC dropped significantly during the validation phase (0.66–0.8 and 0.65–0.71 in the internally and externally validated studies, respectively). The manuscript highlighted methodologic shortcomings and inadequate consideration of electronic automation as significant limitations to successful implementation. In 2019, a similarly styled review identified comparable issues with currently published predictive strategies (47). Interestingly, this study highlighted the fact that much AKI in adults is community acquired which cannot be addressed using most EHR-enabled prediction models. While this is true in adult populations, pediatric AKI tends to be hospital rather than community acquired (48, 49). Thus, it is possible that pediatric populations will benefit more substantially from predictive models.

To give you a better sense of how big data predictive techniques can be applied within the EHR, it may be helpful to discuss an exemplar in greater detail. Tomasev et al. applied deep learning techniques to a US Veteran's Affairs (VA) dataset (50). The dataset consisted of de-identified EHR data for all patients aged 18–90 years who were admitted to a VA hospital between October 2011 and September 2015. In total, the set comprised 703,782 patients and 6,352,945,637 clinical events (individual data elements). Unsupervised, deep learning modeling was applied to this dataset with the goal of predicting AKI. This approach predicted 56% of AKI events and 90% of dialysis-requiring AKI. 84% of Stage 3 AKI was predicted up to 48 h in advance of the event and only two false positive predictions were generated for each true positive. Although this may initially sound like a high false positive rate, responding to all alerts (positive and negative) would require attending to <1% of hospitalized patients. Although this population isn't representative of pediatric inpatients, the technique is certainly applicable and holds great promise. Future efforts should likely utilize similar machine learning methodologies and insure that any final models have the capacity for EHR integration.

## Acute Kidney Injury Alerts

Accurately diagnosing AKI in real time also allows generation of automated notifications or alerts. Simply put, AKI alerts notify care providers as soon as a patient meets the diagnostic criteria for AKI. This information, in turn, allows practitioners to modify care in order to eliminate injurious agents or conditions, prevent progression, and mitigate AKI sequelae (Figure 2). While AKI alerts seem straightforward and effective at first glance, in practice they have proven complex and challenging to implement effectively.

In 2017, Lachance et al. performed a systematic review of AKI alerting studies (51). Six studies comprised of 10,165 patients were included in the analysis. While some of the studies reported improvement in specific care processes, the pooled analysis did not demonstrate improved mortality or a reduced need for renal replacement therapy. Unlike many of the predictive studies described in the above section, the majority of the alerting systems were automated and fully integrated with the EHR. Perhaps the most telling aspect of the studies was the fact that most did not include a clinical decision support component. The studies performed to date are clear on this issue—real time AKI alerting in isolation is inadequate; any such alert must be accompanied by relevant care recommendations.

Since then, several additional alerting studies have been published. Al-Jaghbeer et al. studied 64,512 adult patients with AKI and found that an AKI alert combined with clinical decision support had a significant impact on patient outcomes (52). Although the effect was small, this intervention led to a sustained decreased in length of stay, need for RRT, and mortality. Park et al. studied an alerting mechanism in 3,193 adults (53). In this analysis, the AKI alert was accompanied by an automated nephrology consultation. While they did not find a significant reduction in mortality, they did find that AKI was accurately diagnosed more frequently, the risk for severe AKI was reduced, and AKI recovery was more common. Unfortunately, no outcome driven AKI alerting studies have been performed in children. Holmes et al. did prospectively implement an AKI diagnosis/alerting tool within the national Wales laboratory information management system, however, no intervention was included with the alert (54). As a result, it was not possible to assess the outcome impact of the alert, however, the authors did report a significantly increased incidence of AKI detected by this approach. It is clear that while alerting has great promise, we have not yet fully realized its potential. The combination of an alert with clinical decision support is a large part of the solution, but until better therapeutic options become available, AKI alerts may continue to have only an incremental impact.

## Longitudinal AKI Care and AKI Tracing: The Post-Disease State

Traditionally, AKI was considered a self-limited disease. However, the long-term ramifications associated with renal injury have now been well-described. AKI has been linked with greater risk for new or progressive chronic kidney disease (CKD), hypertension, stroke, and cardiovascular disease (5, 55–58). Despite this, patients who experience AKI often do not receive adequate follow up care (59). Largely, this can be traced to a lack of awareness amongst patients and providers of both AKI and its consequent risks (60). This lack of recognition hampers our ability to track AKI survivors, especially across institutional boundaries and administrative datasets (60, 61).

One of the greatest potential benefits of applying big data concepts to AKI is the ability to overcome many of these barriers. Tracking patients with AKI hinges on our ability to apply an AKI identifier “tag” (61). The aforementioned EHR enabled identification technique described above allows such a tag to be reliably applied. While a myriad of potential identifiers could be used, something as simple as the International Classification of Diseases Ninth/Tenth Revision (ICD-9/10) AKI code might be adequate. Electronically applying the KDIGO AKI definition in an automated fashion within the EHR infrastructure will essentially eliminate the low sensitivity historically associated with ICD9/10 coding (62, 63). Regardless of the tag ultimately chosen, once applied, patients with AKI can be followed at the patient, institution, and population level.

At the patient level, children tagged as AKI survivors could be directed into the appropriate follow up clinic. For example, the AKI tag could, at discharge, automatically notify the primary provider of the diagnosis and place a nephrology referral (64). The discharge order could even generate outpatient orders for creatinine and albumin/creatinine ratios, which is consistent the recommendations of the KDIGO guidelines on AKI (7); currently patients who experience AKI should be assessed within 3 months of the event (7, 25). This is relevant as observational studies have suggested that ambulatory nephrology follow up care after AKI improves outcomes (65). Within an institution, this tag could increase awareness and support clinical decision making. Providers could be directed to avoid nephrotoxic medications or employ more frequent creatinine monitoring in tagged patients. Clearly, at the population and intuitional level, this will be most effective in a self-contained health care organization. Some degree of system integration will be required if follow up care will be provided outside of the institution which applies the tag (61). At the population level, accurate AKI diagnosis and tagging allows patients to be tracked over time. Patients could be assigned a unique identifier which would allow them to be followed between institutions and across administrative databases. The ability to trace patients in this manner would likely lead to a more comprehensive description of the healthcare burden generated by AKI. Administrative databases currently rely upon ICD9/10 codes to identify and track AKI events which is associated with underdiagnosis and a bias toward more severe episodes (60). As a result, the cost and morbidity data based upon analysis of these databases inaccurately reflects of the entire spectrum of disease. Additionally, at the population level, this approach could enable more efficient recruitment into clinical trials and registries which, in turn, creates greater opportunity for scientific advancement.

## Conclusions

Over the past decade, the healthcare community has seen a surge in EHR adoption and the development of innovative informatics methods. Contemporaneously, the critical care and nephrology communities have created a standardized definition for AKI based upon relative chances in discrete data elements. This confluence of events has created unique opportunities for AKI research and care improvement. Integrating the definitional criteria for AKI into the EHR can identify patients who develop AKI precisely at disease onset. This enables the application of predictive models, real time AKI alerting, and tracking of events and patients across institutions, registries, and databases. These interventions, in turn, allow us to better describe AKI epidemiology and improve outcomes at the patient and population level (Figure 2). The promise of EHR enabled big data approaches to AKI discovery and care improvement are substantial and the potential benefits warrant additional work to overcome existing challenges and barriers.

## Author Contributions

SS conceptualized and wrote the manuscript.

### Conflict of Interest

The author declares that the research was conducted in the absence of any commercial or financial relationships that could be construed as a potential conflict of interest. The handling editor declared [DA] a past co-authorship with the author [SS].
